# SGLT‐2 Inhibitors are Associated With a Lower Risk of Multiple Myeloma

**DOI:** 10.1111/ejh.70274

**Published:** 2026-07-29

**Authors:** Enze Liu, Anna Sun, Yuedi Yang, Jing Xu, Attaya Suvannasankha, Brian A. Walker, Pengyue Zhang

**Affiliations:** ^1^ Myeloma Institute, Sylvester Comprehensive Cancer Center, Miller School of Medicine, University of Miami Miami Florida USA; ^2^ Department of Biostatistics and Health Data Science Indiana University Indianapolis Indiana USA; ^3^ Melvin and Bren Simon Comprehensive Cancer Center, Division of Hematology and Oncology, School of Medicine Indiana University Indianapolis Indiana USA

## Abstract

**Aims:**

We aimed to investigate drug response of sodium‐glucose transport protein‐2 inhibitors (SGLT2i) in multiple myeloma (MM), as SGLT2i have potential favorable effects on conditions related to MM.

**Methods:**

We conducted a pharmacoepidemiologic study with a US nationwide health insurance claim data using Cox models to investigate the comparative risk of MM between SGLT2i exposure and dipeptidyl peptidase‐4 inhibitors (DPP4i) exposure. Additionally, we conducted differential gene expression analysis with drug responses in cell lines.

**Results:**

We observed SGLT2i compared with DDP4i exposure was associated with a reduced risk of MM in the general population (hazard ratios [HRs] ≤ 0.37, *p* < 0.001); and subpopulations defined by age, gender, race, deficiency anemia, renal failure, obesity, normal weight, and patients with monoclonal gammopathy of undetermined significance (HRs ≤ 0.49, *p* ≤ 0.032). Moreover, we revealed 450 and 660 unique differentially expressed genes (DEGs) associated with SGLT2i and DPP4i, respectively. We identified that MM‐associated DEGs were significantly enriched in unique DEGs of SGLT2i compared with DPP4i (odds ratio = 2.0, *p* = 0.03). We found consistent DEGs between SGLT2i vs. control and normal vs. MM patients (e.g., AKT1, PHLPP2, CFD, FBLN2 proteasome genes, etc.) that regulate cell cycle, ubiquitination‐proteasome system (UPS), and cell adhesion activities. SGLT2i showed potential beneficial effects in preventing MM, which could be due to SGLT2‐mediated tumor‐suppressing activities for MM from various aspects.

**Conclusion:**

SGLT2i is associated with a lower risk of MM in patients with diabetes.

## 1 Introduction

Sodium‐glucose transport protein‐2 inhibitors (SGLT2i) have beneficial and potential favorable effects on multiple conditions (e.g., type‐2 diabetes [1, 2], obesity [1, 2], chronic kidney disease [3, 4], and cardiovascular disease [3, 4]) that have been linked to the development and progression of multiple myeloma (MM) [5, 6, 7].

In meta‐analyses of clinical trials, SGLT2i had neutral associations with cancer regardless of cancer types [8, 9], and a nonsignificant risk of hematologic and lymphatic malignancies (relative risk = 1.44, 95% confidence interval [CI]: 0.99–2.10) [10]. In population‐based studies, SGLT2i compared with dipeptidyl peptidase‐4 inhibitors (DPP4i) was associated with a lower risk of hematological malignancies in a Hungarian cohort (hazard ratio [HR] = 0.50, 95% CI: 0.28–0.88) [11], and a lower risk of leukemia in a Taiwanese cohort (HR = 0.72, 95% CI: 0.56–0.95) [12]. However, drug response of SGLT2i in MM remains unclear [5].

Gene expression data have been derived from SGLT2i‐/DPP4i‐treated cell lines and patients with MM. On one hand, the Library of Integrated Network‐based Cellular Signatures (LINCS) program revealed drug responses of SGLT2i and DPP4i in transcriptomic profiles of cell lines [13]. On the other hand, the CoMMpass dataset and Zhan et al. [14] offered transcriptome profiles for patients with MM and/or MM‐related conditions. Despite these datasets can be integrated to identify novel drug targets for MM, literature on the relationship involving transcriptome, SGLT2i, and MM is still sparse.

We investigated the relationship between SGLT2i and MM by analyzing real‐world health data and transcriptome data (Figure 1). First, we evaluated the comparative risk of MM between SGLT2i exposure and DDP4i exposure using US nationwide health insurance claims data. We identified that SGLT2i exposure was associated with a reduced risk of MM compared with DDP4i exposure. Second, we conducted differential gene expression analysis by using SGLT2i‐treated, DPP4i‐treated, and untreated cell lines, as well as patients with and without MM. By integrating differentially expressed genes from different sources, we identified that SGLT2i compared with DDP4i was enriched with MM‐associated DEGs, including genes that played tumor suppressing roles in MM.

**FIGURE 1:**
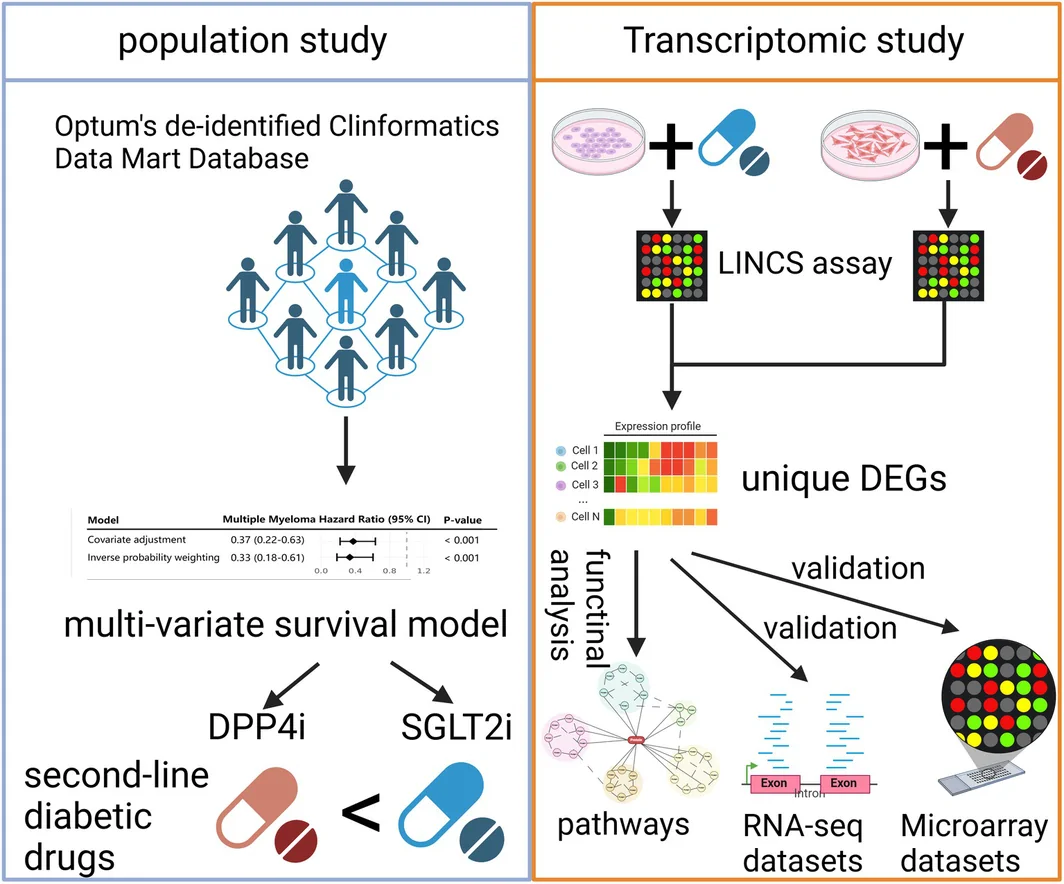
Study workflow.

## 2 Methods

### 2.1 Real‐World Data Source

We used Optum's de‐identified Clinformatics Data Mart Database (Clinformatics). Clinformatics is derived from a database of administrative health claims for members of large commercial and Medicare Advantage health plans. Available data elements included demographic variables (e.g., sex, year of birth, and race), history of health insurance enrollment, diagnosis codes, and pharmacy claims, and so forth. The Indiana University Institutional Review Board (IRB) designated this study as exempt.

### 2.2 Pharmacoepidemiologic Study Design, Outcome, and Covariates

We used active comparator, new user design by selecting DPP4i as the comparator for SGLT2i (Figure S1). We included new initiators of SGLT2i (e.g., dapagliflozin, canagliflozin, empagliflozin and ertugliflozin) or DPP4i (e.g., sitagliptin, saxagliptin, alogliptin and linagliptin) between years 2013 and 2021. We defined the initiation date (e.g., day‐0) as the first date of exposure of SGLT2i or DPP4i. We excluded individuals that had both SGLT2i and DPP4i exposure on or prior to day‐0, only 1‐day of exposure, and missing gender; as well as individuals with at least one of the following conditions prior to day‐0: (1) < 365 days of enrollment, (2) no diabetes, (3) age < 50 years, and/or (4) solid tumor without metastasis, lymphoma, and/or metastatic cancer (Figure S2).

We defined a MM event as the presence of ≥ 2 MM‐related ICD codes (e.g., ICD‐9: 203.0* [multiple myeloma] and ICD‐10: C90.0* [multiple myeloma]) within 1 person‐year, and the corresponding MM event date as the first MM diagnosis date. We defined the outcome as the time from day‐0 to MM event date. We defined the censoring date as the earliest of (1) discontinuation of exposure (e.g., the last date of exposure without exposure in the next 60 days), (2) concurrent exposure to both SGLT2i and DPP4i, or (3) discontinuation from enrollment.

We collected covariates on demographics including age, gender and race. Additionally, we used the R comorbidity package [15] to define covariates on comorbidity including alcohol use disorder, blood loss anemia, cardiac arrhythmias, congestive heart failure, coagulopathy, chronic pulmonary disease, deficiency anemia, depression, diabetes with complication, fluid and electrolyte disorders, hypertension status (nonhypertension, hypertension without complication, and hypertension with complications), hypothyroidism, liver disease, obesity, other neurological disorders, paralysis, pulmonary circulation disorders, psychoses, peptic ulcer disease excluding bleeding, peripheral vascular disorders, renal failure, rheumatoid arthritis or collaged vascular disease, substance use disorder, valvular disease, and weight loss. Moreover, we used ICD codes to define monoclonal gammopathy of undetermined significance (MGUS; ICD‐9273.1 [monoclonal paraproteinemia] and ICD‐10 D47.2 [monoclonal gammopathy]). Additionally, we collected antidiabetic medication exposure‐related covariates including glucagon‐like peptide‐1 receptor agonists (GLP1ra; e.g., exenatide, liraglutide, lixisenatide, albiglutide, dulaglutide, and semaglutide), insulin, metformin, sulfonylureas (e.g., chlorpropamide, glimepiride, glipizide, tolazamide, and tolbutamide), thiazolidinedione (e.g., pioglitazone and rosiglitazone), and other antidiabetic medications (e.g., acarbose, miglitol, bromocriptine, nateglinide, pramlintide, and repaglinide).

### 2.3 Pharmacoepidemiologic Analyses

We used Cox models to estimate hazard ratios (HRs), 95% confidence intervals (CIs), and *p*‐values (*p*). First, we conducted analysis on all individuals. The Cox model was adjusted by all covariates defined in the “pharmacoepidemiologic study design, outcome and covariates” section, and stratified by the index year of day‐0. Second, we conducted a sensitivity analysis utilizing inverse probability of treatment weighting using the propensity score. We used logistic regression model to estimate the propensity score by controlling all covariates in the main analysis and the index year of day‐0. The Cox model included exposure status of SGLT2i/DPP4i and used inverse probabilities of treatment as weights. Third, we analyzed subgroups defined by gender, race (Black and Hispanic), age, heart disease (cardiac arrhythmias or congestive heart failure), deficiency anemia, renal failure, normal weight (i.e., without obesity and weight loss), obesity (without weight loss), and MGUS, which is the precursor condition of MM. The Cox models were adjusted by all covariates in the main analysis excluding the corresponding subgroup‐specific covariates (e.g., excluding gender in gender‐based subgroup analyses), and stratified by the index year of day‐0. All pharmacoepidemiologic analyses were conducted in R.

### 2.4 Gene Expression Data Sources and Processing

We assembled three types of gene expression data (Table 1): (1) cell line expression data, (2) perturbation expression data, and (3) patient expression data.

**TABLE 1:** Dataset summary.

Dataset	Category	Type	Sample size (*N*)	Annotation
LINCS	Perturbation	Expression Assay	16 897	Cell‐lines
DepMap	Cell‐line exp	RNA‐seq	1517	Cell‐lines
CoMMpass	Patient exp	RNA‐seq	837	NDMM; RRMM
GSE114816	Patient exp	RNA‐seq	3	Normal BMPCs
GSE110486	Patient exp	RNA‐seq	5	Normal BMPCs
GSE5900	Patient exp	Affymetrix microarray	78	Normal BMPCs; MGUS; SMM
GSE4452	Patient exp	Affymetrix microarray	65	NDMM

For cell line expression data, we collected RNA‐seq of 19 MM and 1498 non‐MM cell lines from DepMap [13] (expression public 24Q2). We aligned the reads to HG38 genome using STAR [16], and measured gene‐level expression in transcript‐per‐million (TPM [17]) using Gencode V35 annotation with Salmon [18] (quasi‐mode mapping, “validate map” mode). We stabilized the variance with log_2_(TPM + 1) transformation [19].

For perturbation expression data, we collected quantile‐normalized expression values of 257 SGLT2i‐perturbed (canagliflozin, dapagliflozin, empagliflozin, and ertugliflozin) cell lines, 413 DPP4i‐perturbed (alogliptin, linagliptin, saxagliptin, and sitagliptin) cell lines, and 16 229 control (DMSO) cell lines from LINCS [20] dataset (2020 version, https://clue.io/releases/data‐dashboard). These data were normalized and re‐scaled to values between 0 and 8. We subsequently log_2_‐transformed the data.

For patient expression data, we utilized RNA‐seq from 8 normal bone marrow plasma cells (BMPCs, GSE110486 and GSE114816), and 837 patient samples from the CoMMpass cohort [21] including 754 newly diagnosed MM (NDMM) and 83 relapsed and refractory MM (RRMM) samples (accession number phs000748 in the dbGAP database). Here we adopted the same approach for preprocessing cell line expression data to establish gene‐level expression. We applied quantile normalization to remove batch effects. In another dataset with 22 normal BMPCs, 44 monoclonal gammopathy of unknown significance (MGUS), and 65 NDMM samples measured by Microarray (Affymetrix HG‐U133 + 2.0, GSE5900 and GSE4522), we obtained probe‐level expression readout from the GEO database and performed quantile normalization to remove batch effects.

Additionally, we collected gene expression of patients with other cancer types and of healthy organs from the Cancer Genome Atlas (TCGA) and the Genotype‐Tissue Expression [22] (GTEx) databases via GEPIA2 [23]; as well as 918 MM‐associated genes (*p* < 0.05) from the Comparative Toxicogenomics (CTD) database [24].

### 2.5 Gene Expression Data Analysis

For cell line expression data, we used independent *t*‐test to compare the expression of the only DrugBank‐documented [25] SGLT2i‐targeted gene (*SLC5A2*) between MM and non‐MM cell lines in DepMap [13].

For perturbation expression data in LINCS [20], we used LIMMA [26] to identify differentially expressed genes (DEGs) between SGLT2i‐perturbed cell lines and control (DSMO) cell lines, as well as between DPP4i‐perturbed cell lines and control (DSMO) cell lines. Due to the different scales of expression level in the LINCS dataset, we applied elbow rules and empirically used FDR = 1 × 10^−50^ and 1 × 10^−75^ as the cut‐offs for identifying significant DEGs from the two comparisons.

For patient expression data, we used LIMMA [26] to identify DEGs between normal BMPCs and all patient samples from the CoMMpass dataset. We computed the log_2_‐fold change (log_2_(FC)) by using normal BMPCs as the referent. Second, we used LIMMA [26] to identify DEGs between normal BMPCs and MGUS, as well as normal BMPCs and SMM (Table 1). We computed log_2_(FC) by using SMM as the referent. For both analyses, we selected significant DEGs with |log_2_(FC)| > 0.485 and FDR < 0.05.

### 2.6 Gene Essentiality Data Analysis

We obtained the gene essentiality results (CERES [27] scores) of whole‐genome CRISPR knockout experiments in 18 MM cell‐lines from the DepMap database [13]. We defined scores < −1.0, −1.0 ≤ scores ≤ −0.5 and scores > −0.5 as strong, significant, and no inhibition [27], respectively.

### 2.7 Pathway Analysis

Gene Set Enrichment Analysis [28] (GSEA) was conducted for DEGs to reveal pathway/functional changes between conditions. Hallmark pathways, Kyoto encyclopedia of genes and genomes [29] (KEGG) pathways, and Gene ontology [30] (GO) terms were used as pathway annotations. KEGG data are licensed to Dr. Enze Liu and colleagues.

## 3 Results

### 3.1 SGLT2i Exposure Compared With DPP4i Exposure Was Associated With a Reduced Risk of MM

We identified 108 285 individuals with SGLT2i exposure and 147 284 individuals with DDP4i exposure. Among all individuals, those with SGLT2i exposure compared with DDP4i exposure were more likely to be younger (46.8% vs. 33.6% for being 50–64 years), be obese (51.6% vs. 39.3%), have prior GLP1ra exposure (23.3% vs. 6.1%), and have prior insulin exposure (31.9% vs. 21.3%) (Table 2).

**TABLE 2:** Demographics of the study population.

	SGLT2i	DPP4i
*N*	108 285	147 284
Age: %		
50–64	46.8%	33.6%
≥ 65	53.2%	66.4%
Gender: %		
Male	55.2%	48.6%
Female	44.8%	51.4%
Race: %		
White	61.4%	55.9%
Black	13.6%	15.3%
Hispanic	15.4%	18.2%
Asian	3.8%	5.4%
Unknown	6.0%	5.2%
Medication: %		
GLP‐1 receptor agonists	23.3%	6.1%
Insulin	31.9%	21.3%
Metformin	84.3%	82.5%
Sulfonylureas	42.6%	48.1%
Thiazolidinedione	13.1%	13.6%
Other	2.3%	2.4%
Comorbidities: %		
Alcohol use disorder	3.4%	3.2%
Blood loss anemia	2.7%	3.5%
Cardiac arrhythmias	29.2%	29.9%
Congestive heart failure	20.0%	20.6%
Coagulopathy	6.3%	7.0%
Chronic pulmonary disease	32.0%	33.1%
Deficiency anemia	12.8%	15.6%
Depression	28.0%	26.9%
Drug abuse	5.3%	4.6%
Fluid and electrolyte disorders	23.9%	28.3%
Hypothyroidism	25.4%	27.5%
Liver disease	17.6%	15.3%
Obesity	51.6%	39.3%
Other neurological disorders	8.5%	11.9%
Paralysis	1.9%	3.0%
Pulmonary circulation disorders	6.7%	6.9%
Psychoses	2.1%	3.8%
Peptic ulcer disease, excluding bleeding	2.6%	3.0%
Peripheral vascular disorders	26.1%	29.4%
Renal failure	23.2%	31.1%
Rheumatoid arthritis/collaged vascular disease	10.2%	10.3%
Valvular disease	19.8%	20.7%
Weight loss	6.0%	8.5%
Diabetes, complicated	78.9%	72.2%
No hypertension	8.0%	7.5%
Hypertension, complicated	27.5%	30.1%
MGUS	0.5%	0.6%

SGLT2i exposure compared with DPP4i exposure was associated with a reduced risk of MM in all individuals in main (i.e., covariate‐adjusted Cox model) and sensitivity (i.e., inverse probability of treatment weighting using propensity score) analyses (hazard ratios [HR] ≤ 0.37, *p* < 0.001 [Figure 2A]). The 5‐year incidence were 0.1% for SGLT2i exposure and 0.4% for DPP4i exposure in all individuals (log‐rank test *p* < 0.001 [Figure S3]). Additionally, SGLT2i exposure compared with DPP4i exposure was associated with a reduced risk of MM in subpopulations defined by age (50–64 and ≥ 65 years), gender (female and male), Black race, deficiency anemia, renal failure, normal weight, and obesity (HRs ≤ 0.49, *p* ≤ 0.032, Figure 2B). More importantly, SGLT2i exposure led to substantially reduced risk for patients with monoclonal gammopathy of undetermined significance (MGUS), which was the precursor condition of MM (HR = 0.06, *p* = 0.013, Figure 2B). Full results of the Cox model on all individuals were presented in Table S1.

**FIGURE 2:**
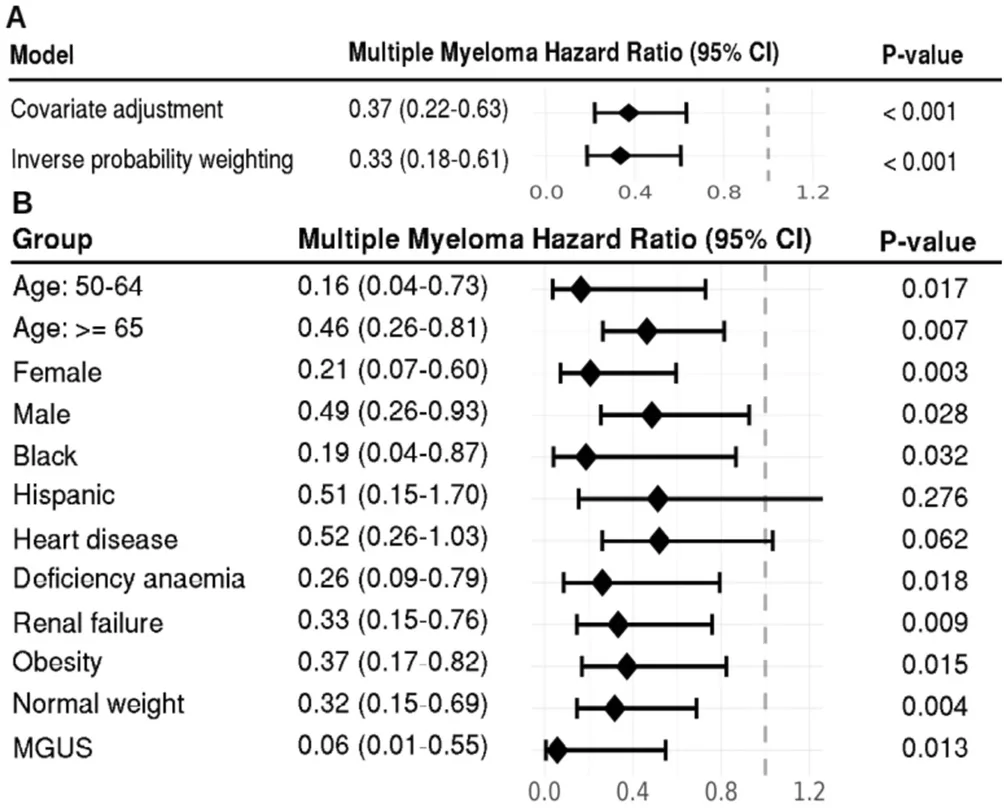
Hazard ratios of sodium‐glucose transport protein‐2 inhibitors (SGLT2i) compared with dipeptidyl peptidase‐4 inhibitors (DDP4i). (A) Associations with multiple myeloma in all individuals. (B) Associations with multiple myeloma in subpopulations.

### 3.2 SGLT2i Induced Significant Transcriptomic Changes Regardless of *SLC5A2* Expression

In cell line expression data (i.e., DepMap [13]) analysis, we identified *SLC5A2* (the only SGLT2i‐targted gene according to DrugBank [25]) exhibited a minimal expression (i.e., og_2_(TPM + 1) < 1) in most cell lines, and was not differently expressed between MM and non‐MM cell lines (Figure 3A). In analysis of expression profiles in TCGA and GTEx databases, we also observed *SLC5A2* had a low expression in almost all cancer types and healthy organs except kidney, bone and testis (Figure S4). Additionally, *SLC5A2* was identified nonessential among 18 MM cell‐lines documented in DepMap [13] (median CERES [27] score −0.04, Figure S5).

**FIGURE 3:**
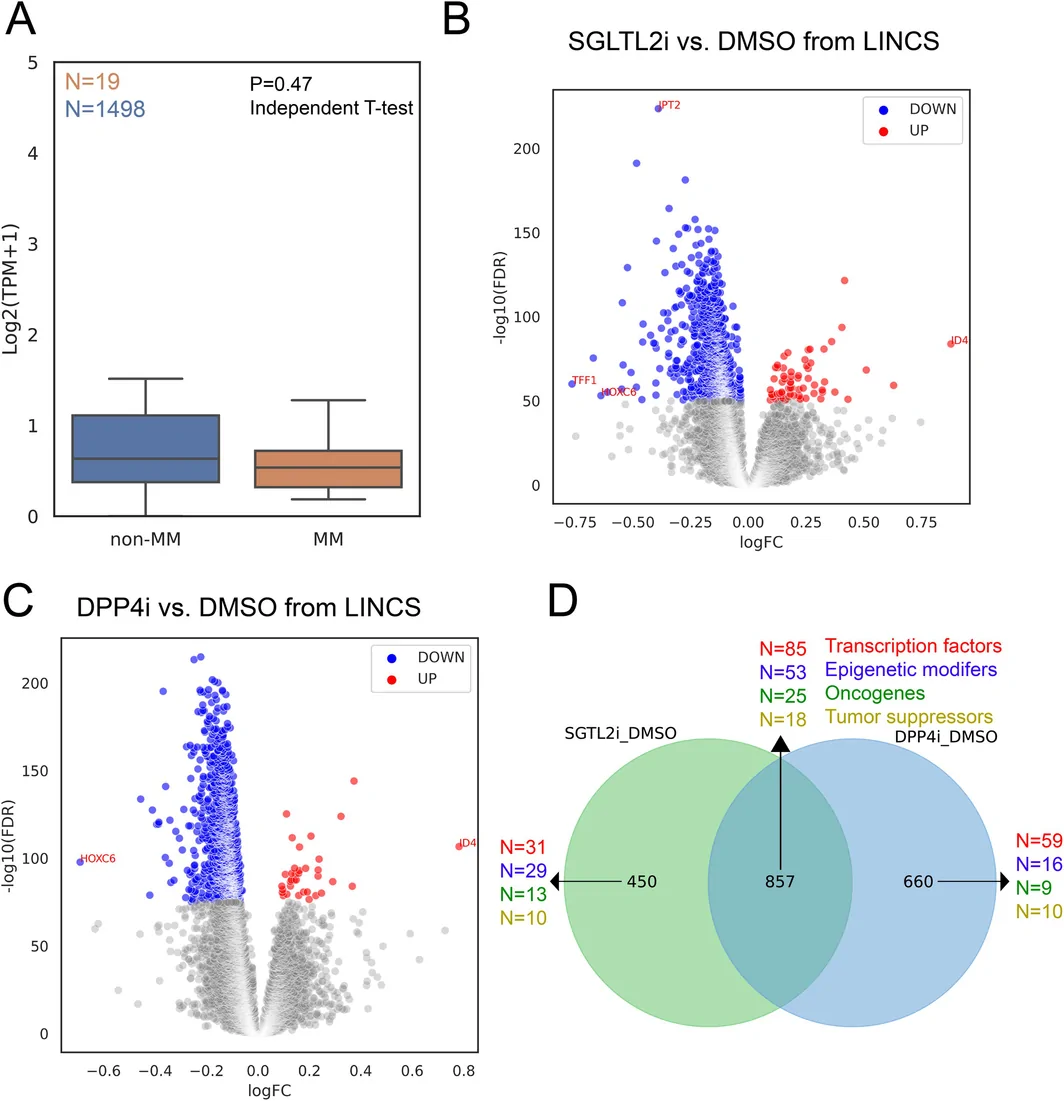
Transcriptomic change of tumor cell‐lines before/after SGLT2i and DPP4i perturbation. (A) Expression of *SLC5A2* (the target of SGLT2i) among MM and non‐MM cell‐lines. Volcano plots indicated significant DEGs found between SGLT2i and DMSO cell‐lines (B), and DPP4i and DMSO cell‐lines (C) documented in LINCS dataset. (D) Common and unique significant DEGs found between two comparisons.

In perturbation expression data (i.e., LINCS [20]) analysis, we used all tumor cell lines due to limited number of MM cell line (*N* = 1) in the data. We selected 1 × 10^−50^ and 1 × 10^−75^ under the elbow rule as cutoffs of FDR in DEG analysis for STGL2i and DPP4i (Figure S6), respectively. We identified 1307 DEGs for SGLT2i vs. DMSO and 1517 DEGs for DPP4i versus DMSO, several of which were common DEGs (Figure 3B,C). Of these, we identified 450 DEGs (52 up‐ and 398 down‐regulations) unique to SGLT2i and 660 DEGs unique to DPP4i (Figure 3D).

### 3.3 Unique Perturbation Signatures Brought SGLT2i Tumor Suppressing Advantages Over DPP4i

We retrieved 918 MM‐associated genes (*p* < 0.05) from the Comparative Toxicogenomics (CTD) database [24] and mapped them onto DEGs unique to SGLT2i or DPP4i. We observed MM‐associated genes were approximately two times more enriched in DEGs unique to SGLT2i compared with DPP4i (odds ratio = 2.0, Fisher exact test *p* = 0.03).

In functional analysis for DEGs unique to SGLT2i, we identified a list of potential MM‐related genes. We observed *CDF* (a hallmark of MM [31] and obesity‐associated gene [32]) was among the top down‐regulated genes (log_2_FC = −0.67, Figure 4A). Additionally, we found *NRP1* (a key regulator in angiogenesis in MM [33]), *AKT1*, *MAPKAPK2*, and *MK2* (genes associated with MM proliferation and/or progression [34, 35]) were also among the top‐ranked dysregulated genes.

**FIGURE 4:**
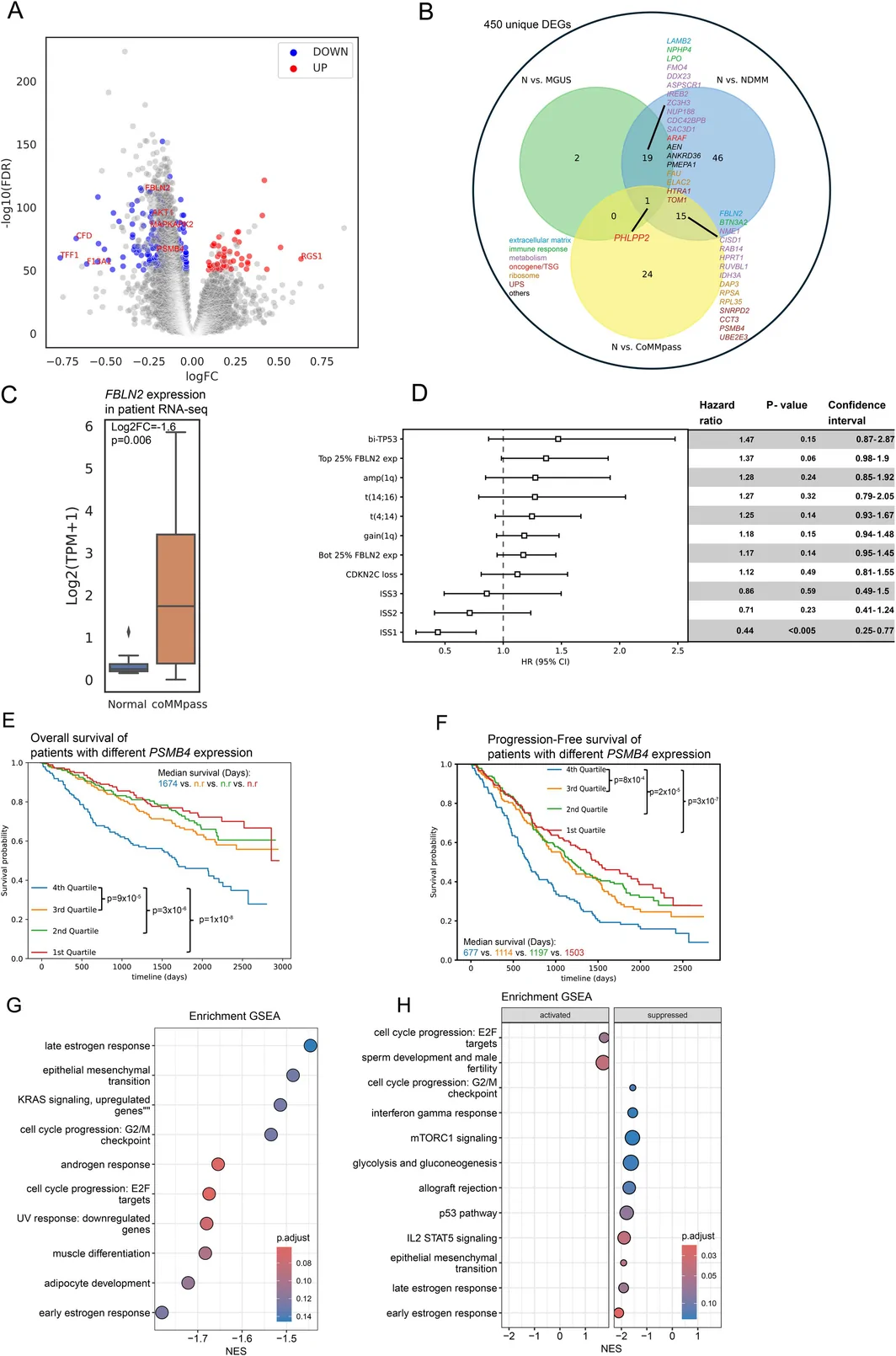
Expression of 450 unique DEGs in validation sets. (A) a volcano plot indicating 450 significant DEGs existed in SGLT2i but not in DPP4i. (B) A Venn diagram indicating the consistency of 450 unique DEGs in validation sets. (C) *FBLN2* expression in the validation dataset. (D) Multivariate survival analysis results for patients in NDMM population of MMRF cohort with different expression of *FBLN2*. Kaplan–Meier curves indicating the PFS (E) and OS (F) difference of patients with different *PSMB4* expression. (G) GSEA analysis results indicating top‐ranked significantly dysregulated hallmark pathways derived from 450 unique DEGs. (H) GSEA analysis results from 1517 significant DEGs revealed between DPP4i vs. DMSO.

### 3.4 DEGs Unique to SGLT2i Overlapped With MM‐Specific DEGs

We identified a few DEGs unique to SGLT2i were overlapped with MM‐specific DEGs in patient expression data analysis (Figure 4B). First, we observed *PHLPP2* (a tumor suppressing gene [36]; log_2_FC = 0.14 and FDR = 1 × 10^−52^ in perturbation expression data analysis) was consistently dysregulated in MM patients (log_2_FC = 0.63, 0.89, and 0.96, and FDR = 6 × 10^−4^, 6 × 10^−8^, and 3 × 10^−4^, for normal BMPCs vs. MGUS, and CoMMpass, respectively). Additionally, in SGLT2i‐purturbed cell lines, we found *PHLPP2* was negatively correlated (ρ = −0.32, Pearson correlation) with *AKT1*, of which high expression was significantly associated with inferior progression free survival (*p* < 0.01, log‐rank test, Figure S7A) and overall survival (*p* < 0.08, Figure S7B) in NDMM population of the CoMMpass dataset. Second, we observed *FBLN2* (a gene encoding the extracellular matrix protein (Fibulin‐2) and associated with MM microenvironment [37]; log_2_FC = 0.3 and FDR = 1 × 10^−115^ in perturbation expression data analysis) was found consistently over‐expressed in MM in the validation set (log_2_FC = 1.6, *p* = 0.006, Figure 4C). In multivariate analysis, we further found high expression of *FBLN2* was a marginally significant risk factor (HR = 1.4, *p* = 0.06) for disease progression among all well‐known risk factors (Figure 4D).

Moreover, as the hallmark of MM, Ubiquitination Proteasome system (UPS) plays a critical role in maintaining protein homeostasis in MM cells [38]. Among five UPS‐related genes, three (*PSMB4*/*TOM1*/*CCT3*) demonstrated a significant association with PFS and OS (*p* < 0.05, log‐rank test), respectively. MM Patients with consistently high expression of three genes (samples consistently in top 50% expression of three genes vs. samples in bot 50%) reflected a significantly inferior survival compared to the rest (*p* = 0.01 and 0.02 for PFS and OS, respectively, Figure S8A,B), while patients with other 33 cancer types in TCGA didn't reflect such significant association (*p* = 0.1 and 0.9, Figure S9A,B, obtained from GEPIA2 [23]). Among them, *PSMB4* and *CCT3* demonstrated strong inhibition among all MM cells (Median CERES [27] score −2.2 and−1.8, Figure S10). *CCT3* was previously found to undergo a gradually increase from normal BMPCs to MM, and from MUGS, SMM, MM to plasma cell leukemia (PCL) [39]. *PSMB4* encodes a subunit of proteasome and was extensively reported to be associated with MM proliferation and progression [40]. Among 754 NDMM patients in CoMMpass dataset, its risk elevated along with the expression (*p* < 2 × 10^−5^, Figure 4E,F).

We also conducted GSEA analysis using 450 unique DEGs against hallmark pathways (Figure 4G), leading to 10 significant (FDR < 0.25) pathways that were all downregulated. After comparing dysregulated pathways obtained from GSEA analysis using all DEGs from DPP4i (Figure 4H) with Figure 4G, we identified pathways such as “cell cycle progression,” “KRAS signaling,” “E2F targets,” and “UV response downregulated genes” as the unique features of SGLT2i.

## 4 Discussion

We identified sodium‐glucose transport protein‐2 inhibitors (SGLT2i) exposure compared with dipeptidyl peptidase‐4 inhibitors (DPP4i) exposure to be associated with a reduced risk of multiple myeloma (MM) in US individuals with health insurance. Additionally, we observed MM‐associated differentially expressed genes (DEGs) were enriched in DEGs unique to SGLT2i compared with DPP4i in perturbation expression data analysis. Moreover, we found a list of DEGs unique to SGLT2i that were related to the cellular machinery of MM in functional analysis (e.g., *CDF*, *AKT1*, *MAPKAPK2*, and *MK2*), or associated with clinical outcomes of MM in MM patient expression data analysis (e.g., *PHLPP2* and *FBLN2*).

We derived new knowledge on drug response of SGLT2i in MM using a large US cohort. We observed SGLT2i vs. DPP4i exposure was consistently associated with a lower risk of MM (0.06 ≤ hazard ratios [HRs] ≤ 0.49, *p* ≤ 0.032) in covariate‐adjusted Cox model and Cox model with inverse probability of treatment weighting using propensity score, as well as in subpopulations defined by age, gender, Black race, heart disease, deficiency anemia, renal failure, obesity, normal weight and MGUS patients (Figure 2). Previous studies found metformin [41, 42] and glucagon‐like peptide‐1 (GLP‐1) agonists receptor agonists [43] were associated with protective effects against MM, while these studies were not based on a setting of time from drug initiation to the development of MM. One study using a US veteran cohort shown a potential beneficial effect of SGLT2i on MM, while the study could be underpowered due to limited sample size [44].

We also revealed the cellular mechanisms between SGLT2i and MM. We found SGLT2i dramatically regulated the cellular machinery even with the low expression level of its only documented target *SLC5A2* [25] (Figures 3 and 4). More importantly, we identified unique perturbation signatures of SGLT2i (e.g., *CDF*, *AKT1*, *MAPKAPK2*, *MK2*, *PHLPP2*, and *FBLN2*) that provided tumor suppressing roles and led to a reduced risk of developing MM compared to DPP4i. Expression of *CFD* has been reported not only as a hallmark that differentiate MM from other cancers in a pan‐cancer blood plasma proteomics study (*N* = 1800) [31], but also associated with obesity [32]. *AKT1* can be regulated by signals from microenvironment such as cytokines [34] which supports MM proliferation and progression in many aspects [34]. *MAPKAPK2* and *MK2* participates in numerous cellular processes such as stress response and inflammatory response [45]. These genes work in concert with AKT kinases to promote MM proliferation [35].

We observed that SGTL2i brough potential protective effect both intra‐ and extra‐cellularly. Intra‐cellularly, we observed that *PHLPP2* underwent consistent downregulation in all MM samples compared to normal among all datasets in this study while SGTL2i upregulated its expression in malignant cells. As a well‐known negative regulator of the tumor master regulator *AKT1*, *PHLPP2* mediates increased apoptosis and inhibition of proliferation by dephosphorylating the hydrophobic motifs of AKT isoforms [46, 47], while AKT signaling pathway regulates a wide range of cellular processes including survival, proliferation, metabolism and mobility, including MM [48]. Although *PHLPP2* was not extensively studied in MM, our findings suggested its important role in stopping normal plasma cells from turning into MM cells, possibly via inhibiting *AKT1* and its signaling pathway. Moreover, SGLT2i enhanced the DNA damage repair function, regulated cell cycle and UPS activity, the last of which was a hallmark and a unique vulnerability of MM cells. Extracellularly, SGLT2i inhibited the extracellular matrix and cell adhesion activity, possibly via *FBLN2*. *FBLN2* was recognized as an oncogene in solid tumors [49]. Its spiked expression was previously reported between NDMM and MGUS/normal BMPCs cells [50] although the reason was not unveiled. MM cells adhered to bone marrow stromal cells to survive and triggered the secretion of growth factors such as IL‐6, IGF‐1, and VEGF [51], in which *FBLN2* expression might serve as a critical step. Our results suggested the potential role of *FBLN2* expression in regulating tumorigenesis and disease progression. Additionally, we validated these unique features with multiomics patient data and confirmed them were MM‐specific.

We observe SGLT2i can alter gene expression despite its known target SLC5A2 being minimally expressed in MM cells and in most tissues outside the kidney. Such an observation leads us to hypothesize that these gene expression changes come from off‐target (i.e., off‐SLC5A2) effects. First, it is plausible that there are unknown targets of SGLT2i in MM cells that drive the suppression of MM, such as ubiquitin‐proteasome system and the AKT1/PHLPP2 signaling axis. SGLT2i, as a small molecule, may also interact with other proteins, and affect the corresponding signaling pathways. Secondly, the deregulation of extracellular matrix and cell adhesion genes suggested the potential disruption of critical interaction between MM cells and bone marrow microenvironment. We expect additional multiomics data analyses to provide more mechanistical insights.

Our study has limitations. First, as the real‐world health data are derived from individuals with diabetes and with commercial and/or US Medicare Supplemental health insurance plans, our pharmacoepidemiologic analyses results may not be generalized to other populations. Second, our pharmacoepidemiologic analyses are subject to unmeasured confounding effects related to weight, body mass index (BMI), blood glucose level, insulin sensitivity, environmental exposure, genetic mutations, and especially socioeconomic status, as SGLT2i vs. DPP4i is newer and more expensive. Additionally, as SGLT2i vs. DPP4i is often prescribed to patients with a higher risk of cardiovascular conditions and/or existing renal conditions, our pharmacoepidemiologic analyses are therefore subject to healthy user and/or indication bias, as well as bias related to frequency of medical follow‐up. For instance, SGLT2i vs. DPP4i users may have a higher frequency of medical follow‐up due to a potential higher socioeconomic status and/or risk of cardiovascular disease, which may relate to the early detection of MM. Third, the outcome, exposure, and covariates could be misclassified in administrative health claim data. We largely assume the true clinical outcomes can be represented by health insurance claims. Fourth, the analyses are subject to potential model misspecification. For instance, in the covariate‐adjusted Cox model, some covariates may not satisfy the proportional hazard assumption. We use both the covariate‐adjusted Cox model and the Cox model using inverse probability of treatment weighting to improve the confidence of the result. Last, due to the lack of perturbed MM cell lines in the LINCS dataset, we utilized all cancer cells perturbed by two inhibitors and identified the most significant molecular features that represented the common pan‐cancer features. Given these limitations, our observations should not be interpreted as causality but rather as associations deserving subsequent validations. Nonetheless, our study examined the potential benefit in a large real‐world dataset and provided a potential molecular mechanism that accounted for such benefit using drug response data and patient omics data. This will provide new opportunities for the discovery on how to effectively prevent MM for individuals with diabetes.

## Author Contributions

E.L. and P.Z. conceptualized the study. E.L., A.S., P.Z., Y.Y., J.X., A.S., and B.A.W. participated in study design, data preparation, interpretation of results, and/or the critical writing and editing of the manuscript. E.L., A.S., and P.Z. also participated in the data analyses.

## Funding

The authors have nothing to report.

## Ethics Statement

The authors have nothing to report.

## Consent

The authors have nothing to report.

## Conflicts of Interest

The authors declare no conflicts of interest.

## Supporting information


**Figure S1:** Illustration of the pharmacoepidemiologic study design.
**Figure S2:** Flowchart of the pharmacoepidemiologic study cohort.
**Figure S3:** Cumulative incidence of multiple myeloma following sodium‐glucose transport protein‐2 inhibitors (SGTL2i) or dipeptidyl peptidase‐4 inhibitors (DPP4i) exposure.
**Figure S4:** Normalized gene expression level of SLC5A2 among all cancer types documented and their normal control listed in TCGA.
**Figure S5:** Gene essentiality (CERES) scores of 18 MM cell‐lines from the whole‐genome CRISPR knockout experiment data in DepMap.
**Figure S6:** A line plot indicating the ranked FDR calculated from differential expression analysis on LINCS data.
**Figure S7:** Survival difference of NDMM patients with different AKT1 expression level in PFS (A) and OS (B).
**Figure S8:** Survival difference of NDMM patients with different CCT3/TOM1/PSMB4 overall expression level in PFS (A) and OS (B).
**Figure S9:** Survival difference of cancer patients in TCGA dataset with different CCT3/TOM1/PSMB4 overall expression level in PFS (A) and OS (B).
**Figure S10:** Gene essentiality (CERES) scores of three UPS‐coding genes among 18 MM cell‐lines in DepMap.
**Table S1:** Associations with multiple myeloma: covariate‐adjusted Cox model using all individuals.

## Data Availability

Clinformatics Data Mart Database is available from Optum (optum.com). LINCS Level‐3 data (2020 version) are available from CLUE (https://clue.io/releases/data‐dashboard). Summary statistics are included within the article and/or supporting materials.
