# Supplementary material for: SGLT‐2 Inhibitors are Associated With a Lower Risk of Multiple Myeloma
Source: Eur J Haematol. 2026 Jul 29;117(5):1347–57. doi: 10.1111/ejh.70274 (PMC13643622; doi:10.1111/ejh.70274)
Supplement: Supplementary file 1 [file EJH-117-1347-s001.pdf]

SGLT-2 Inhibitors Are Associated with a Lower risk of Multiple Myeloma

Enze Liu,[1 *] Anna Sun,[2] Yuedi Yang,[2] Jing Xu,[2] Attaya Suvannasankha[3], Brian A. Walker,[1]

Pengyue Zhang[2 *]

1. Myeloma Institute, Sylvester Comprehensive Cancer Center, Miller School of Medicine, University of Miami, Miami, Florida, 33027, United States of America
2. Department of Biostatistics and Health Data Science, Indiana University, Indianapolis, Indiana 46202, United States of America
3. Melvin and Bren Simon Comprehensive Cancer Center, Division of Hematology and Oncology, School of Medicine, Indiana University, Indianapolis, Indiana 46202, United States of America

* Correspondence authors

Enze Liu: exl1075@miami.edu

Pengyue Zhang: zhangpe@iu.edu

SUPPLEMENTARY FIGURE LEGENDS

Supplementary Figure 1. Illustration of the pharmacoepidemiologic study design.

Supplementary Figure 2. Flowchart of the pharmacoepidemiologic study cohort.

Supplementary Figure 3. Cumulative incidence of multiple myeloma following sodium-glucose transport protein-2 inhibitors (SGTL2i) or dipeptidyl peptidase-4 inhibitors (DPP4i) exposure.

Supplementary Figure 4. Normalized gene expression level of *SLC5A2* among all cancer types documented and their normal control listed in TCGA.

Supplementary Figure 5. Gene essentiality (CERES) scores of 18 MM cell-lines from the whole-genome CRISPR knockout experiment data in DepMap.

Supplementary Figure 6. A line plot indicating the ranked FDR calculated from differential expression analysis on LINCS data.

Supplementary Figure 7. Survival difference of NDMM patients with different *AKT1* expression level in PFS (A) and OS (B).

Supplementary Figure 8. Survival difference of NDMM patients with different *CCT3*/*TOM1*/*PSMB4* overall expression level in PFS (A) and OS (B).

Supplementary Figure 9. Survival difference of cancer patients in TCGA dataset with different *CCT3*/*TOM1*/*PSMB4* overall expression level in PFS (A) and OS (B).

Supplementary Figure 10. Gene essentiality (CERES) scores of three UPS-coding genes among 18 MM cell-lines in DepMap.

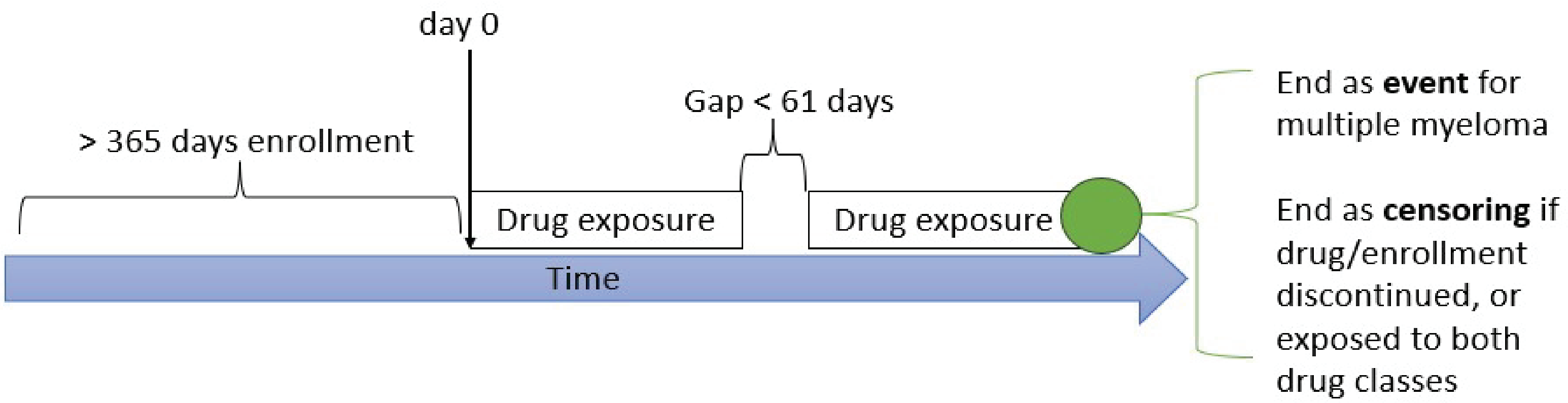

Supplementary Figure 1. Illustration of the pharmacoepidemiologic study design.

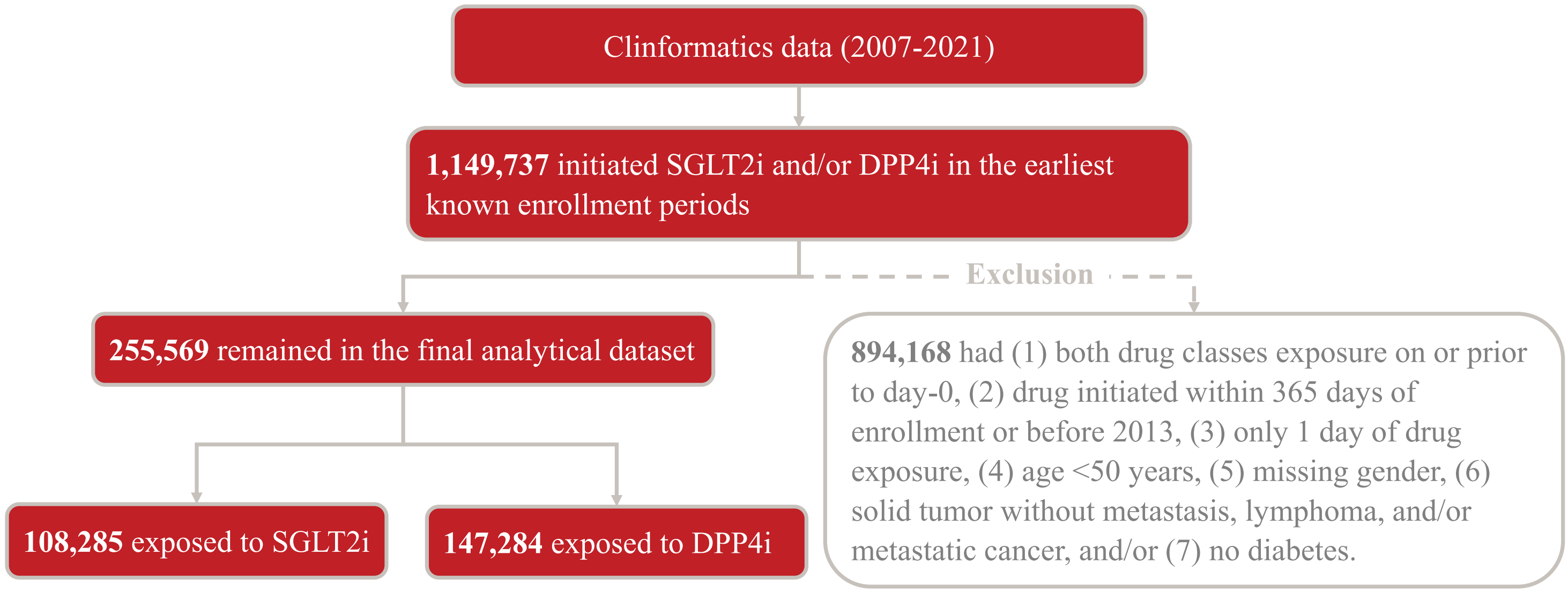

SGLT2i: sodium-glucose transport protein-2 inhibitors. DPP4i: dipeptidyl peptidase-4 inhibitors

Supplementary Figure 2. Flowchart of the pharmacoepidemiologic study cohort.

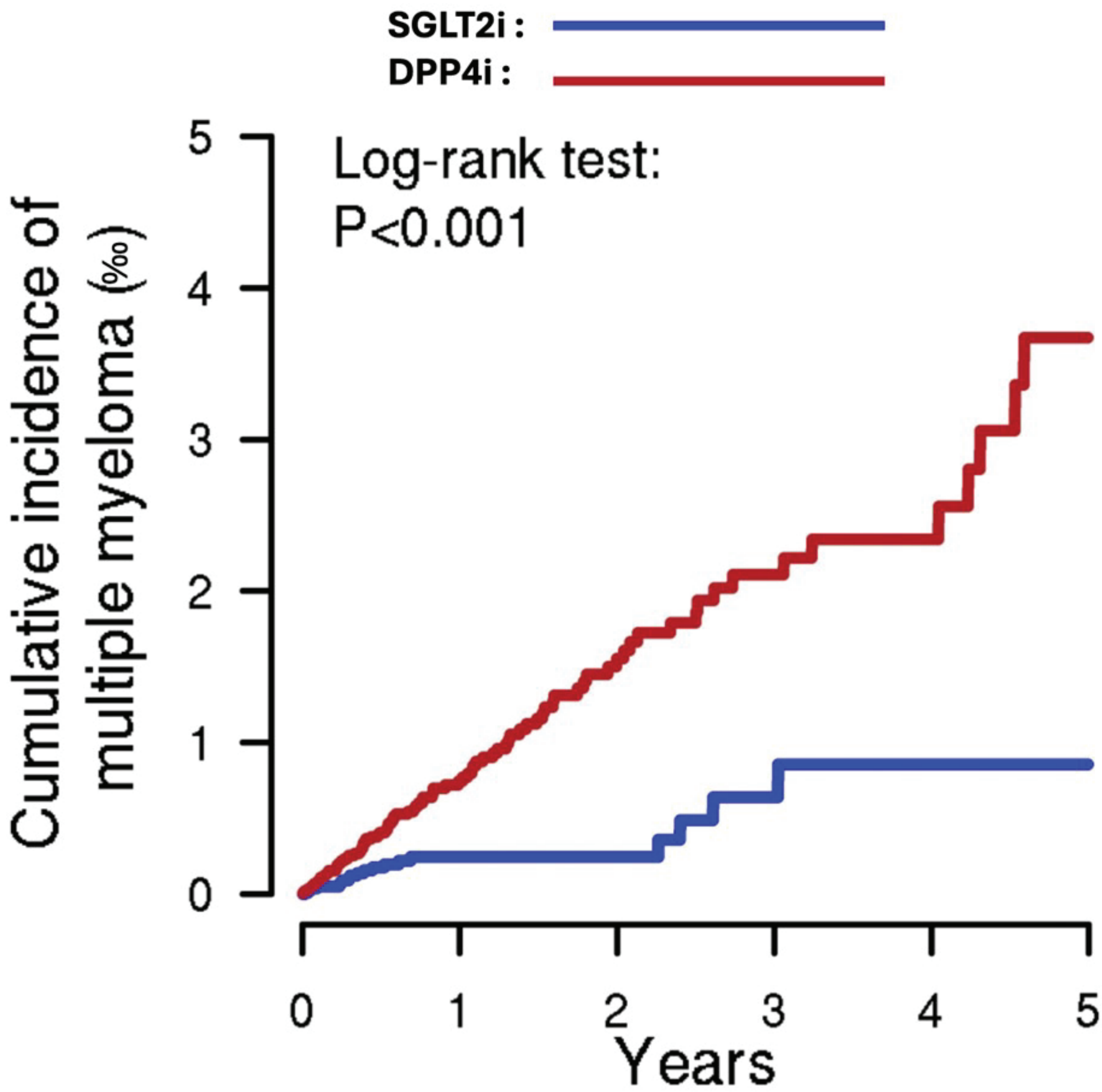

Supplementary Figure 3. Cumulative incidence of multiple myeloma following sodium-glucose transport protein-2 inhibitors (SGLT2i)or dipeptidyl peptidase-4 inhibitors (DPP4i) exposure.

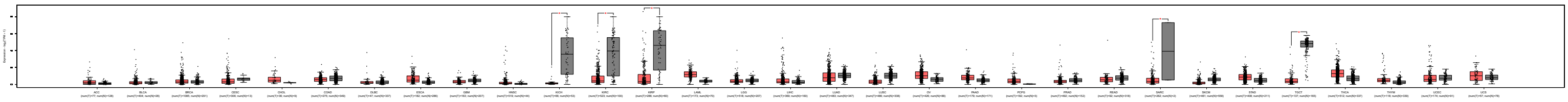

Supplementary Figure 4. Normalized gene expression of *SLC5A2* among all cancer types and their normal control listed in TCGA.

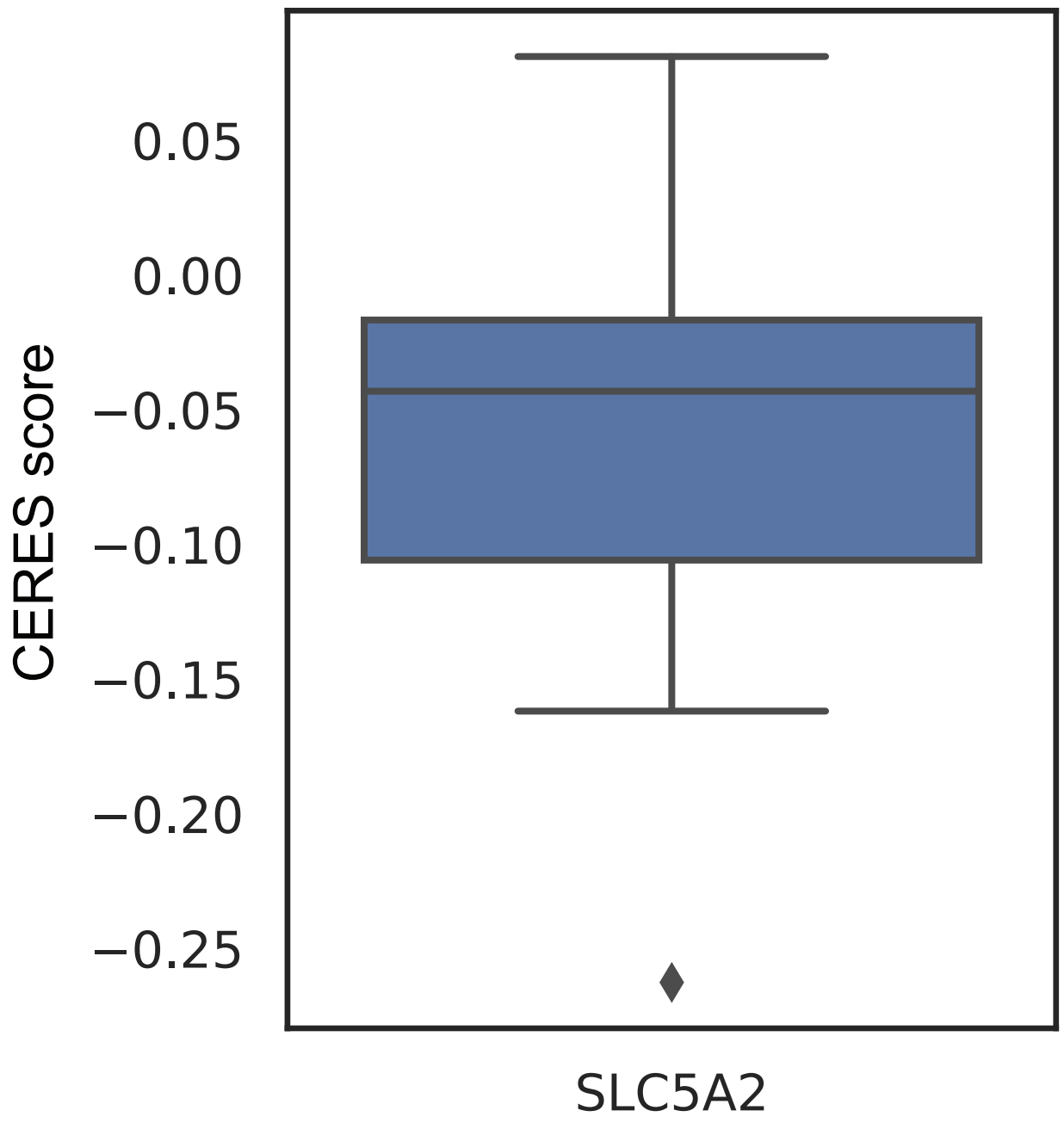

Supplementary Figure 5. Gene essentiality (CERES) scores of 18 MM cell-lines from the whole-genome CRISPR knockout experiment data in DepMap.

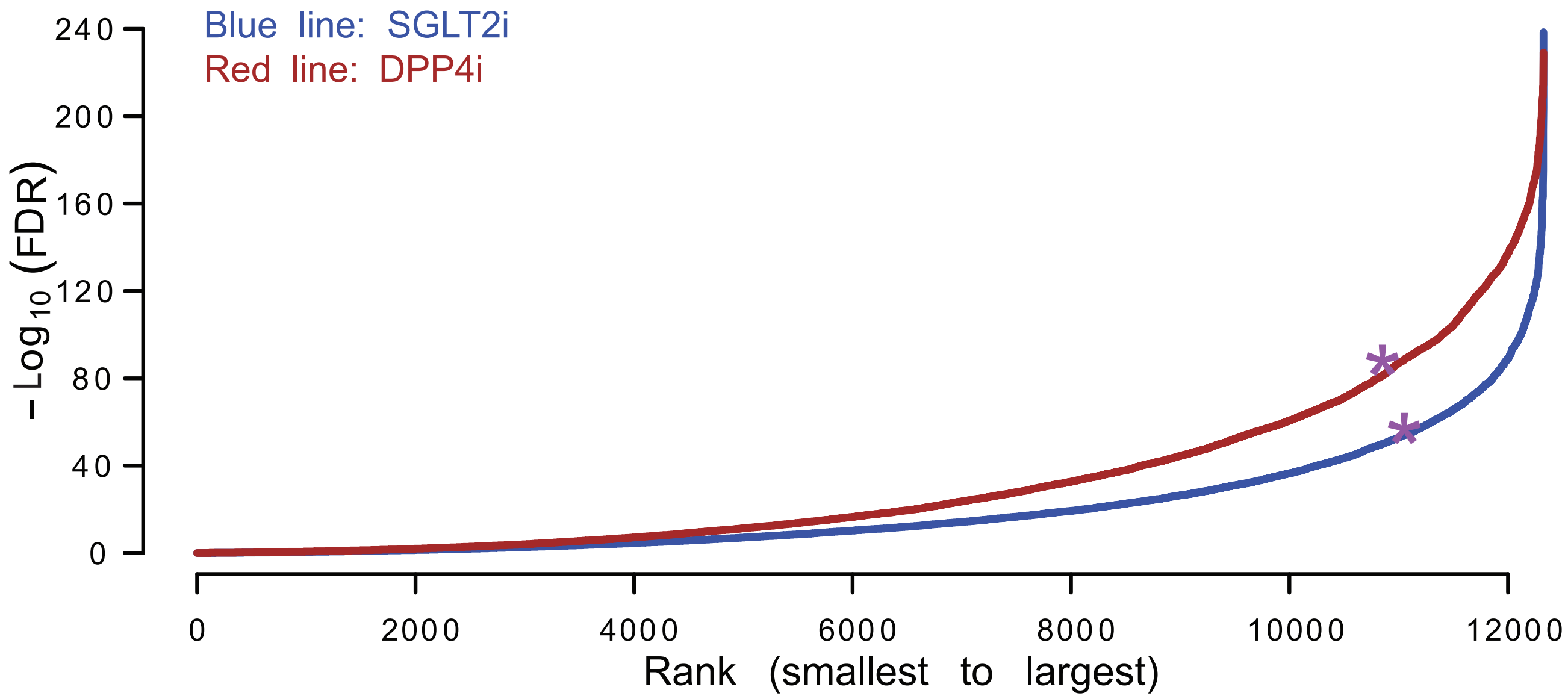

Supplementary Figure 6. A line plot indicating the ranked FDR calculated from differential expression analysis on LINCS data

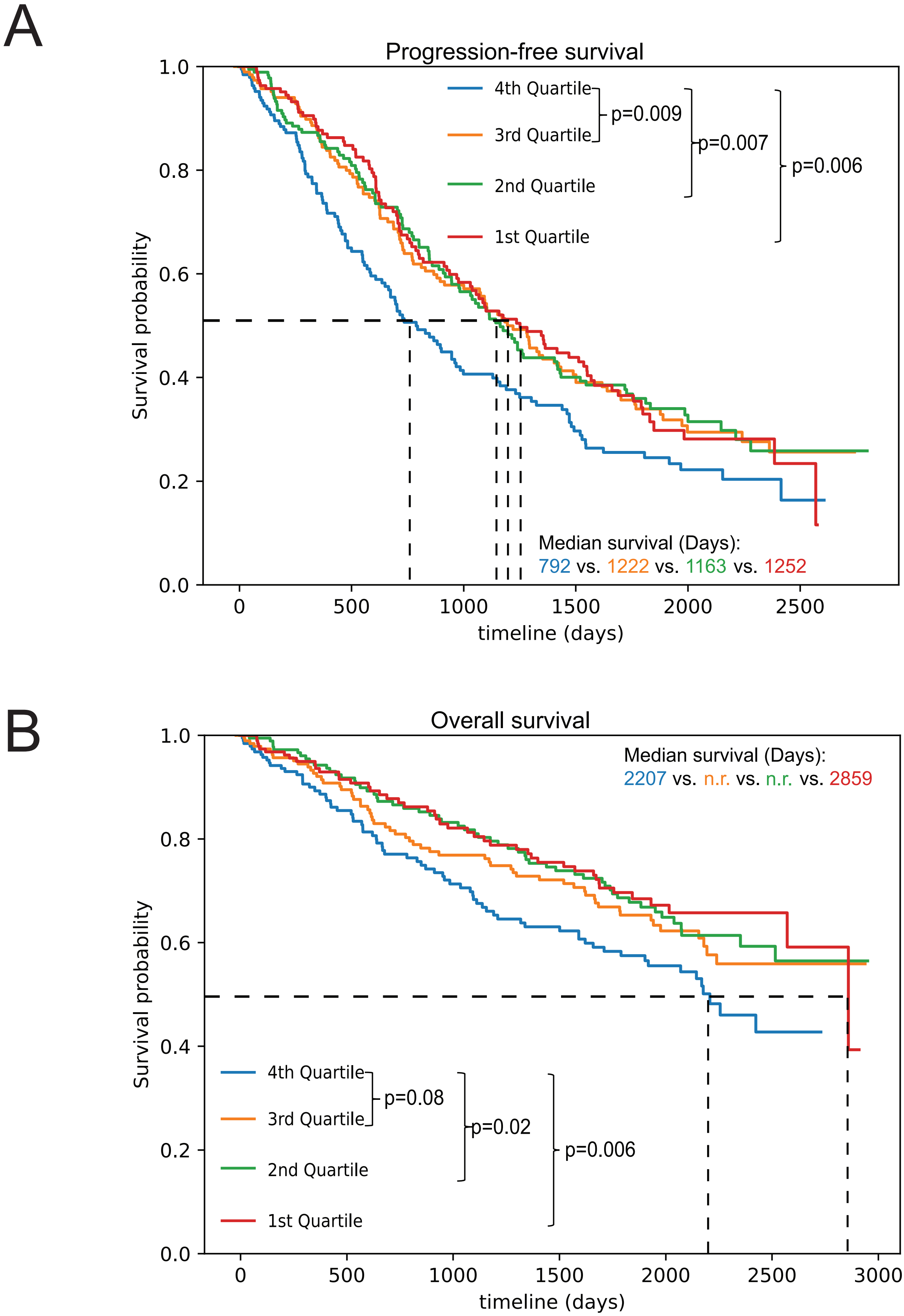

Supplementary Figure 7. Survival difference of NDMM patients with different *AKT1* expression level in PFS (A) and OS (B).

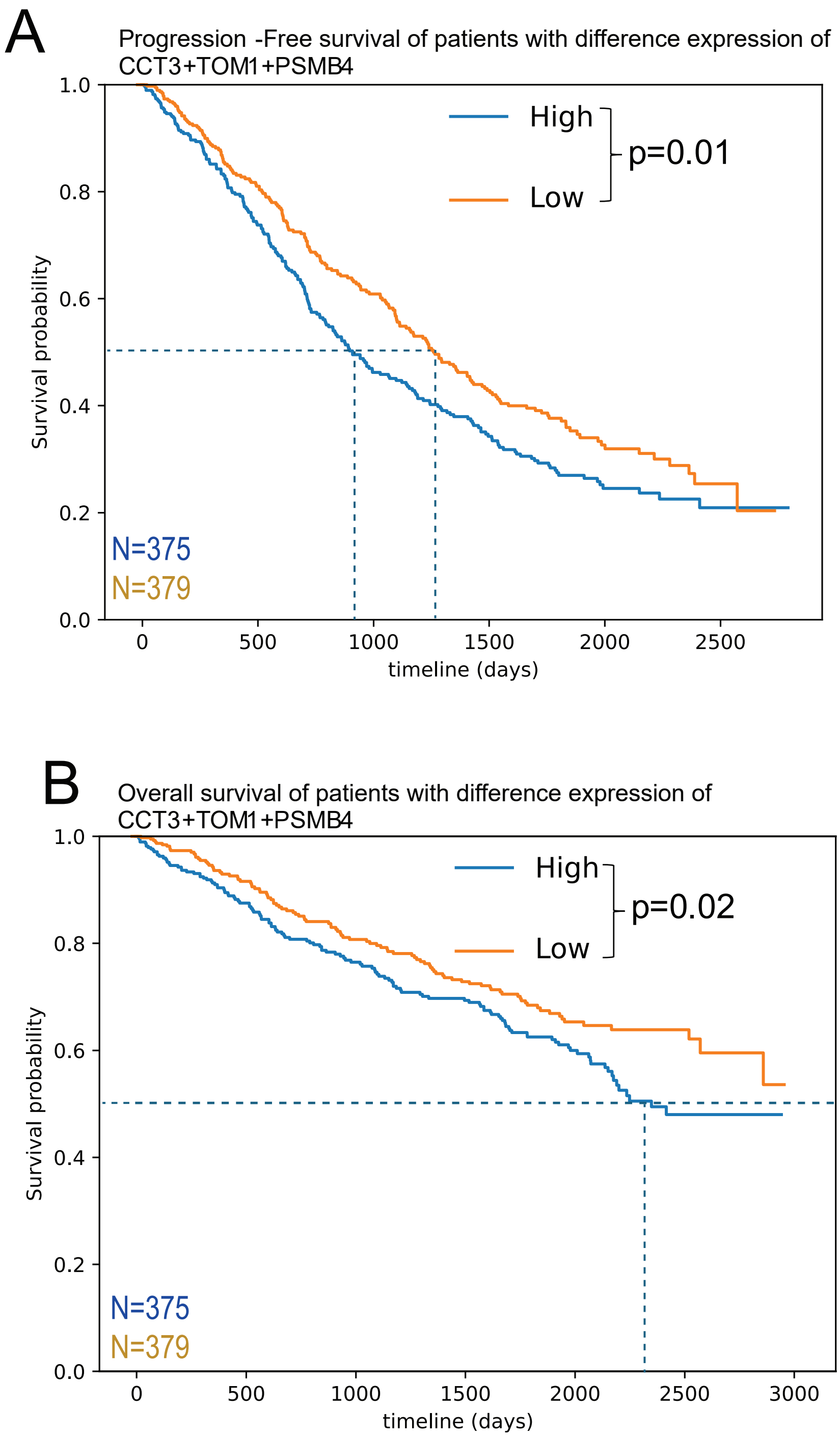

Supplementary Figure 8 Survival difference of NDMM patients in CoMMpass dataset with different *CCT3/TOM1/PSMB4* overall expression level in PFS (A) and OS (B).

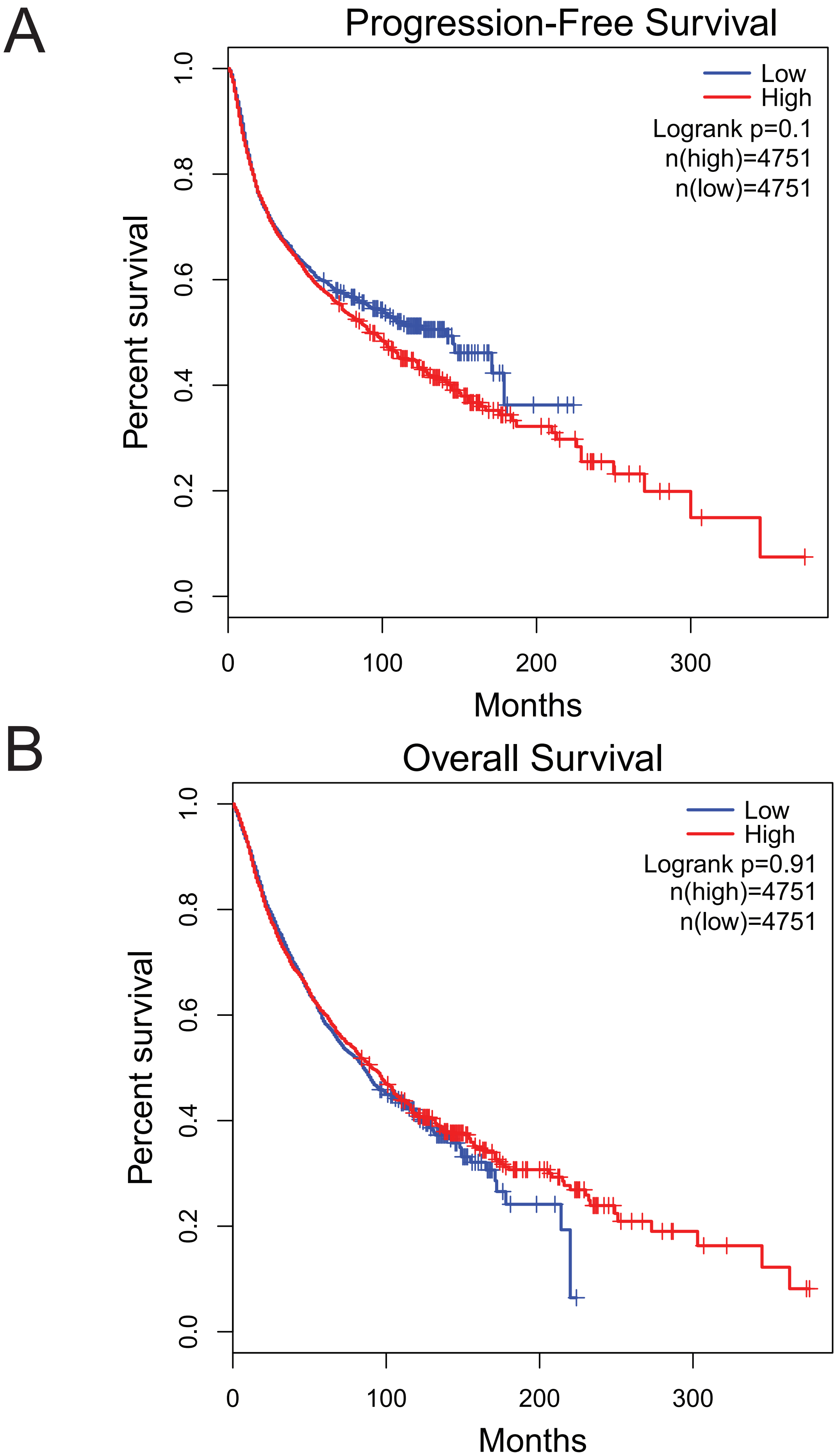

Supplementary Figure 9 Survival difference of cancer patients in TCGA dataset with different *CCT3/TOM1/PSMB4* overall expression level in PFS (A) and OS (B).

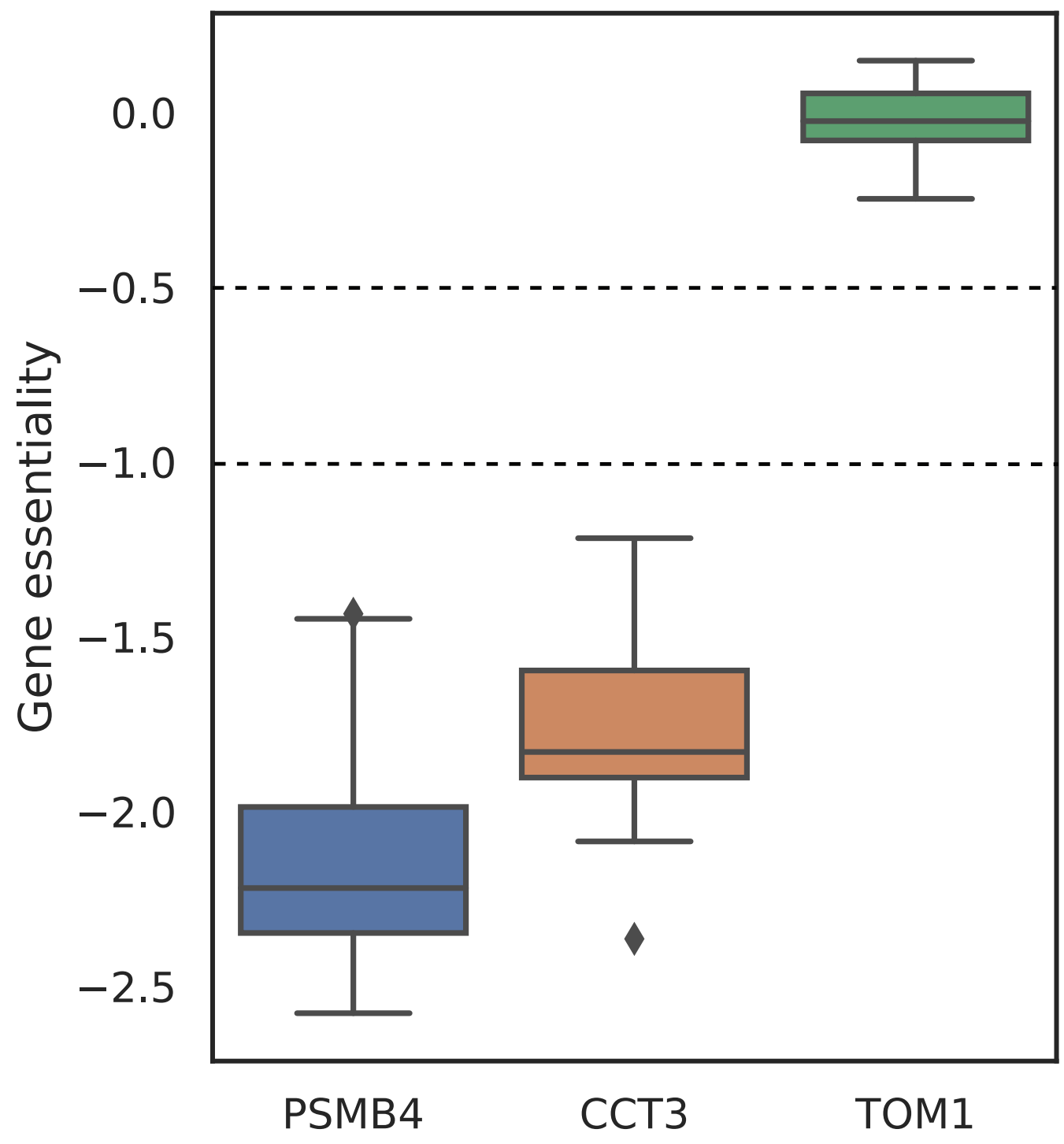

Supplementary Figure 10. Gene essentiality (CERES) scores of three UPS-coding genes among 18 MM cell-lines in DepMap.

SUPPLEMENTARY TABLE LEGENDS

Supplementary Table 1. Associations with multiple myeloma: covariate-adjusted Cox model using all individuals.

Supplementary Table 1. Associations with multiple myeloma: covariate-adjusted Cox model using all individuals.

Variables	HR (95% CI)	P
**SGLT2i vs. DPP4i**	0.37 (0.22-0.63)	<0.001
**Age** (referent:50-64)		
≥65	2.39 (1.43-4.00)	<0.001
**Gender** (referent: Male)		
Female	0.79 (0.54-1.15)	0.223
**Race** (referent: White)		
Black	1.69(1.05- 2.71)	0.030
Hispanic	1.28(0.78- 2.10)	0.333
Asian	1.37(0.64- 2.90)	0.416
Unknown	1.47(0.67- 3.24)	0.336
**Comorbidities**		
Alcohol abuse	1.10(0.40- 3.07)	0.850
Blood loss anemia	0.37(0.11- 1.22)	0.102
Cardiac arrhythmias	1.44(0.95- 2.17)	0.085
Congestive heart failure	0.99(0.61- 1.59)	0.960
Coagulopathy	1.07(0.57- 2.00)	0.843
Chronic pulmonary disease	1.25(0.85- 1.84)	0.258
Deficiency anemia	1.84(1.20- 2.82)	0.005
Depression	0.79(0.50- 1.25)	0.316
Drug abuse	0.56(0.17- 1.80)	0.328
Fluid and electrolyte disorders	1.04(0.68- 1.59)	0.868
Hypothyroidism	1.02(0.68- 1.51)	0.940
Liver disease	1.08(0.67- 1.77)	0.743
Obesity	1.17(0.80- 1.70)	0.424
Other neurological disorders	0.78(0.43- 1.40)	0.399
Paralysis	1.24(0.49- 3.12)	0.655
Pulmonary circulation disorders	0.75(0.37- 1.50)	0.410
Psychoses	1.07(0.42- 2.74)	0.880
Peptic ulcer disease, excluding bleeding	1.14(0.45- 2.85)	0.783
Peripheral vascular disorders	1.03(0.69- 1.54)	0.889
Renal failure	1.62(1.04- 2.53)	0.034
Rheumatoid arthritis/collaged vascular disease	0.99(0.56- 1.75)	0.965
Valvular disease	1.17(0.75- 1.81)	0.493
Weight loss	0.68(0.34- 1.38)	0.287
Diabetes, complicated	1.00(0.62- 1.63)	0.991
No hypertension	0.53(0.16- 1.69)	0.281
Hypertension, complicated	0.74(0.46- 1.18)	0.203
MGUS	12.94(6.92-24.19)	<0.001
**Other medications**		
GLP-1	0.66(0.31- 1.41)	0.287

Insulin	1.46(0.97- 2.21)	0.071
Metformin	1.01(0.64- 1.58)	0.967
Sulfonylureas	0.69(0.48- 1.00)	0.051
Thiazolidinedione	1.19(0.73- 1.94)	0.474
Other antidiabetic medications	2.39(1.15- 4.94)	0.019
